# Postnatal Acute Famine and Risk of Overweight: The Dutch Hungerwinter Study

**DOI:** 10.1155/2012/936509

**Published:** 2012-05-07

**Authors:** Annet F. M. van Abeelen, Sjoerd G. Elias, Tessa J. Roseboom, Patrick M. M. Bossuyt, Yvonne T. van der Schouw, Diederick E. Grobbee, Cuno S. P. M. Uiterwaal

**Affiliations:** ^1^Julius Center for Health Sciences and Primary Care, University Medical Center Utrecht, P.O. Box 85500, 3508 GA Utrecht, The Netherlands; ^2^Department of Clinical Epidemiology, Biostatistics and Bioinformatics, Academic Medical Center, University of Amsterdam, Meibergdreef 9, 1105 AZ Amsterdam, The Netherlands; ^3^Department of Obstetrics and Gynecology, Academic Medical Center, University of Amsterdam, Meibergdreef 9, 1105 AZ Amsterdam, The Netherlands

## Abstract

*Objective*. To examine the association between undernutrition during postnatal periods of development and the risk of overweight in adulthood. *Methods*. We studied 8,091 women from Prospect-EPIC, exposed to the Dutch famine at ages between 0 and 21 years, recruited at ages between 49 and 70 years. We used linear and logistic regression models to explore the effect of famine on BMI, waist circumference, and the risk of overweight. *Results*. Overall, postnatal famine exposure was associated with increased BMI and waist circumference in a dose-dependent manner (*P*  for trend < 0.01). Furthermore, risk of overweight was increased following famine exposure (*P*  for trend = 0.01), with those severely exposed at ages 0–9 years having 25% (95% CI 1.05 to 1.50) higher risk compared to unexposed women. *Conclusions*. This study is the first to directly show a positive association between short and transient undernutrition during postnatal development and BMI, waist circumference, and overweight in adulthood.

## 1. Introduction

Obesity is an increasing problem worldwide; it is the fifth leading risk for death globally. Furthermore, overweight and obesity are major risk factors for chronic diseases, including cardiovascular disease, type 2 diabetes, and cancer [1]. Global estimates of the World Health Organization (WHO) indicate that more than one in ten of the world's adult population was obese in 2008 [1]. Once, obesity was considered a nutritional disease and only a problem in developed countries. However, to date the number of people suffering from overweight and obesity is dramatically increasing in developing countries as well [1]. Worldwide, a total of 43 million children under five were overweight in 2010; more than 80% of these children live in developing countries [1]. Since childhood overweight is an important precursor of overweight and obesity in adulthood [2], these numbers predict increasing fractions of overweight and obese people in the future in both developed and developing countries.

 The developmental origins of chronic disease hypothesis propose that undernutrition during important periods of growth and development, including fetal life, infancy, and childhood, results in early adaptations in the structure and function of the body [3]. These adaptations may be beneficial for survival in the short term. However, in the long term, these adaptations may result in an increased risk of chronic diseases, including obesity, coronary heart disease, and type 2 diabetes.

The association between low birth weight, as a marker of intrauterine growth retardation, postnatal catch-up growth, and later body composition and chronic disease risk has been extensively researched. Humans who suffered from fetal growth retardation and subsequently showed catch-up growth were demonstrated to have higher susceptibility to obesity, type 2 diabetes, and cardiovascular disease in later life [4–9]. Such catch-up growth was also found to be associated with a disproportionate increase in abdominal fat mass [9, 10].

Direct evidence for the early origins of obesity was provided by studies of people who were conceived during the Dutch famine. These studies demonstrated that externally imposed undernutrition during gestation, followed by adequate food supply later on, was associated with an increased risk of obesity [11–13]. Furthermore, the Dutch Famine Birth Cohort Study demonstrated associations between undernutrition during gestation and obesity-related phenotypes in adult life, including an atherogenic lipid profile [14], coronary heart disease [15], and a reduced glucose tolerance [16, 17]. Women exposed to famine in early gestation also had an increased risk of cardiovascular mortality [18].

Overall, this evidence suggests that undernutrition during fetal life, which is an important period of growth and development, is critical with respect to later life health outcomes. Next to the fetal period, childhood and adolescence are also important periods of growth and development. Little if anything is known about the later life effects of exclusive postnatal stunting of growth. Studies of these effects would require registry of exposures in distinct phases of postnatal childhood as well as registry of outcome data in individuals that were born healthy. In the Prospect-EPIC cohort study we have data with individual information on exposure to the 1944-1945 Dutch famine during childhood, adolescence, and young adulthood. In this way, we were able to examine the association between moderate or severe undernutrition during postnatal periods of development—including childhood, adolescence, and young adulthood—and BMI, waist circumference, and the risk of overweight in adult life.

## 2. Subjects and Methods

### 2.1. The Prospect-EPIC Cohort

This study included women participating in Prospect-EPIC, one of the two Dutch contributions to the European Prospective Investigation into Cancer and nutrition (EPIC). The rationale and design of both EPIC and Prospect-EPIC have been described in detail elsewhere [19, 20]. In brief, the Prospect-EPIC study includes 17,357 women living in Utrecht and vicinity, aged 49–70 years at enrolment between 1993 and 1997. All women signed informed consent before study inclusion. The study complies with the Declaration of Helsinki and was approved by the Institutional Review Board of the University Medical Center Utrecht. At baseline, the women filled in a general questionnaire on demographic and lifestyle factors, and past and current morbidity and a food frequency questionnaire, and underwent a brief standardized physical examination. In addition, a nonfasting blood sample was taken.

### 2.2. Famine Exposure

#### 2.2.1. The Dutch Famine

The Dutch famine was a six-month period of severe undernutrition during the last winter of World War II. The famine struck the occupied and densely populated Western parts of The Netherlands. The average daily rations per capita dropped from about 1,400 kilocalories in October 1944 to below 1,000 kilocalories in late November 1944. At the height of the famine from December 1944 to April 1945, the official daily rations varied between 400 and 800 kilocalories, less than a quarter of the prefamine rations [21]. The relative amounts of fats, carbohydrates, and proteins remained essentially unchanged during this period [22]. In early May 1945, The Netherlands was liberated and food supplies became abundant due to Allied intervention, ending the famine abruptly.

#### 2.2.2. Famine Exposure Assessment

The self-administered general questionnaire, which was filled in at time of enrolment by all study participants, contained questions about the 1944-1945 Dutch famine. Women were asked about their place of residence during the 1944-1945 Dutch famine and about their experiences of hunger and weight loss. Women could respond to these last two questions using one of three answer categories: “hardly,” “little,” or “very much.” The women who had answered “not applicable” or “I do not know” to one or both famine questions were excluded from the analysis. We combined the answers into a three-point subjective famine exposure score: women who reported having been “very much exposed” to both hunger and weight loss were categorized as “severely exposed,” women who reported having been “hardly exposed” to either hunger and weight loss were categorized as “unexposed,” and all others as “moderately exposed.”

#### 2.2.3. Exposure Age Categories

Age at famine exposure was assessed taking October 1, 1944 as the start of the famine as reference. Exposure age was classified into three categories; childhood (0 to 9 years of age at famine exposure), adolescence (10 to 17 years of age at famine exposure), and young adulthood (18 years or older at famine exposure). We defined these three general growth periods according to the seven stages in the postnatal human life cycle as defined by Bogin [23]. We defined preadolescent childhood, a period of rapid growth with many developmental milestones in physiology, behavior, and cognition, as the period between 0 and 9 years, just before the growth spurt in women [23, 24]. From the start of the growth spurt, at around 10 years, through to age 17 is called adolescence [23, 24]. This period is characterized by the growth spurt including sexual development [23, 24]. From 18 years of age, we considered persons as young adults gradually reaching homeostasis in physiology.

### 2.3. Subject Selection

The Prospect-EPIC cohort consists of 17,357 women. For the present study, we excluded women who were born after the famine (*n* = 2,559) and women who resided outside occupied Netherlands during the famine (*n* = 1,732). Women for whom no hunger score could be calculated were also excluded (*n* = 4,975), leaving 8,091 women for our analyses. In the models where we adjusted for total energy intake, we also excluded women who were likely to have misreported their energy intake (*n* = 2,451); the definition of energy misreporters is described below.

### 2.4. Outcome Assessment

The baseline physical examination, assessed in light indoor clothing without shoes, was carried out by trained study staff. Body height was measured to the nearest 0.5 cm with a wall mounted stadiometer (Lameris, Utrecht, the Netherlands), body weight was measured to the nearest 0.5 kg with a floor scale (Seca, Atlanta, GA, USA), and waist and hip circumference were measured as the minimal circumference of the middle, respectively, the hip/buttocks, to the nearest 0.5 cm with a nonstretchable measuring tape. Body mass index (BMI) was calculated by dividing weight in kilograms by the square of height in meters (kg/m^2^). Waist to hip ratio (WHR) was calculated by dividing waist circumference in centimeters by hip circumference in centimeters.

### 2.5. Covariates

At baseline, participants completed a general questionnaire containing questions on demographic characteristics, presence of chronic diseases, and risk factors for chronic diseases, such as smoking habits and level of education. Smoking was defined according to the number of pack years. We categorized level of education into low (primary and lower vocational education), intermediate (advanced elementary, intermediate vocational, and higher general secondary education from 3rd year with success or completed), or high (higher vocational education, university to bachelor examination, and university completed) and used it as a proxy for socioeconomic status.

 Daily dietary intake was obtained from a food frequency questionnaire containing questions on the usual frequency of consumption of 79 main food items during the year preceding enrolment. This questionnaire allows the estimation of the mean daily consumption of 178 foods. It has been validated against 12 24 h dietary recalls [25]. The 1996 Dutch food consumption table was used to calculate energy and nutrient intakes. Basal metabolic rate (BMR) was estimated using the Schofield equations [26]. Participants with a total energy intake to BMR ratio of <1.14 or >2.40 were defined as energy misreporters, according to the Goldberg et al. cut-offs [27].

### 2.6. Data Analysis

Prospect participant characteristics at enrolment, including demographics, energy and macronutrient intake, and lifestyle, were first tabulated against timing and severity of famine exposure, in order to evaluate potential for confounding. We used linear regression analysis to explore the effects of famine exposure on BMI and waist circumference separately. To analyze the effect of famine exposure on the risk of overweight we performed logistic regression analyses. We defined a person to be overweight if their BMI was ≥25 kg/m^2^.

To study the effects of famine exposure, we used the three-point individual famine exposure score (unexposed, moderately, and severely exposed). Trend tests were used to explore dose-response relations by introducing the famine exposure score as a continuous variable (1 for “unexposed,” 2 for “moderately exposed,” and 3 for “severely exposed”). To assess sensitive growth periods, we analyzed the effects of famine exposure on BMI, waist circumference, and overweight within each of the exposure age categories (0–9, 10–17, and ≥18 years) and tested for interaction by introducing the cross-product of the famine exposure score and age at start of the famine to the various models.

First we analyzed the crude association between famine exposure and BMI, waist circumference, and overweight in each of the exposure age categories. In a second model, we adjusted for potential confounders including age at start of the famine (years), smoking (pack years), alcohol intake (g/day), and level of education (low/intermediate/high; socioeconomic status proxy). In a subsequent model, we additionally included average total energy intake in the year prior to enrolment as a possible intermediate variable linking childhood undernutrition to later overweight. In the model with total energy intake included, we excluded women who were likely to have misreported their energy intake. Within the women with reliable energy intake, we compared the confounder adjusted model with such model with energy intake added.

Continuous variables were introduced as such in the different models; for categorical variables we created indicator variables. We evaluated the linear regression model assumptions with a normal probability plot of the standardized residuals (normality assumption) and a scatterplot with standardized residuals versus standardized predicted values (constant variance assumption and linearity assumption). These assumptions were found to be justified. Introducing nonlinear terms to the models did not improve the fit of the data. Results of the linear regression analyses are reported as mean differences with 95% confidence intervals (CIs) between those who were moderately or severely famine exposed compared to those who were unexposed to famine. Results of the logistic regression analyses are reported as odds ratios (ORs) with 95% CI between those who were unexposed to famine compared to those who were moderately or severely famine exposed.

We performed all statistical analyses with SPSS Statistics version 17.0 (SPSS, Chicago, IL, USA). *P* values were based on two-sided tests with a cut-off level for statistical significance of 0.05.

## 3. Results

Table 1 shows the baseline characteristics of the study group. Of the total of 8,091 women, 4,425 (55%) women had experienced the famine between ages 0 to 9 years, 3,179 (39%) women at ages between 10 and 17 years, while 487 (6%) women were 18 years or over when they experienced the famine. In total, 3,675 (45%) women reported having been unexposed to famine; 3,078 (38%) had been moderately exposed and 1,338 (17%) severely exposed to famine. Women who were older at the start of the famine reported more often to be exposed to famine. Overall, severely famine exposed women had lower total energy intake and smoked more than unexposed women.

### 3.1. BMI and Waist Circumference

Tables 2 and 3 show the mean differences in BMI and waist circumference for all ages combined and within each of the three exposure age categories (0–9 years, 10–17 years, and ≥18 years) for those who were moderately or severely famine exposed compared to those unexposed to famine. In the three exposure age categories combined, we observed a significantly higher BMI and waist circumference among famine exposed women in a dose-dependent manner (*P* for trend for BMI: 0.002; *P* for trend for waist circumference: <0.001). After adjustment for the potential confounders age at start of the famine, smoking, alcohol intake, and level of education (socioeconomic status proxy), mean differences in BMI were 0.29 kg/m^2^ (95% CI: 0.10 to 0.48) and 0.21 kg/m^2^ (95% CI: −0.05 to 0.46), and mean differences in waist circumference were 0.75 cm (95% CI: 0.28 to 1.23) and 0.65 cm (95% CI: 0.02 to 1.27), for moderate and severe famine exposure, respectively, compared to no famine exposure.

Additionally including total energy intake did not change the associations between famine exposure and both BMI and waist circumference. We found a statistically significant interaction between the effects of age at start of the famine and famine exposure on BMI (*P *for interaction for BMI: 0.02; *P* for interaction for waist circumference: 0.07).

Within women in the 0-to-9 year exposure age category, we found a significant dose-dependent increase in BMI and waist circumference among famine-exposed women compared to unexposed women (*P* for trend BMI and waist circumference: <0.001). After adjustment for the potential confounders age at start of the famine, smoking, alcohol intake, and level of education (socioeconomic status proxy), mean differences in BMI were 0.33 kg/m^2^ (95% CI: 0.07 to 0.59) and 0.48 kg/m^2^ (95% CI: 0.13 to 0.83), and mean differences in waist circumference were 0.84 cm (95% CI: 0.21 to 1.48) and 1.02 cm (95% CI: 0.17 to 1.88), for moderate and severe famine exposure respectively compared to no famine exposure. Further inclusion of total energy intake in the multivariable model did not change the results.

We could not demonstrate a significant association between famine exposure and BMI and waist circumference among women in the 10-to-17 year and ≥18-year exposure age categories. Adjustment for the potential confounders age at start of the famine, smoking, alcohol intake, and level of education did not change the results, as did the additional adjustment for total energy intake.

### 3.2. Overweight

In the three exposure age categories combined, we observed a significantly increased risk of overweight among famine-exposed women in a dose-dependent manner (*P* for trend: 0.001). After adjustment for the potential confounders age at start of the famine, smoking, alcohol intake, and level of education, odds ratios were 1.10 (95% CI: 1.00 to 1.22) and 1.15 (95% CI: 1.01 to 1.31), for moderate and severe famine exposure, respectively, compared to no famine exposure. Additionally including total energy intake did not change these results. The *P* for interaction for the effect of age at start of the famine on the association between famine exposure and the risk of overweight was 0.10.

**Figure 1:**
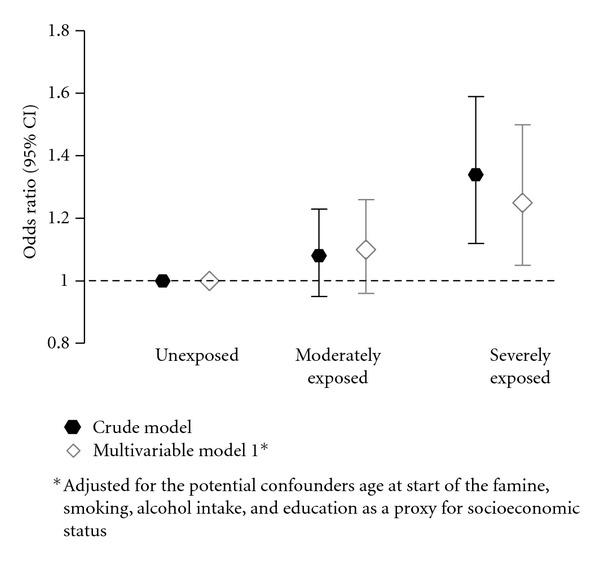
Odds ratios (ORs) and 95% confidence intervals (CI) for the risk of overweight (BMI ≥ 25 kg/m^2^) for women within the 0-to-9-year exposure age category who reported to be moderately or severely exposed to famine compared to those who reported to be unexposed to famine.

Within women aged 0 to 9 years at start of the famine, those moderately famine exposed had no increased risk compared to unexposed women, whereas those severely famine exposed had a significantly increased risk of overweight (*P* for trend: 0.002) (Figure 1). After adjustment for the potential confounders age at start of the famine, smoking, alcohol intake, and level of education, those moderately famine exposed had nonsignificant 10% higher odds (95% CI: 0.96 to 1.26) of overweight, whereas those severely famine exposed had a significant 25% higher odds (95% CI: 1.05 to 1.50) of overweight compared to unexposed women (Figure 1). Further inclusion of total energy intake did not change the risk estimates.

We could not demonstrate a significant association between famine exposure and the risk of overweight among women in the 10-to-17 year and ≥18-year exposure age categories. After adjustment for the potential confounders age at start of the famine, smoking, alcohol intake, and level of education, odds ratios among women in the 10-to-17-year exposure age category were 1.13 (95% CI: 0.96 to 1.33) for moderate and 1.01 (95% CI: 0.82 to 1.25) for severe famine exposure compared to no famine exposure. Among women in the ≥18-year exposure age category, the adjusted odds ratios were 1.15 (95% CI: 0.75 to 1.78) for moderate and 1.60 (95% CI: 0.89 to 2.87) for severe famine exposure compared to no famine exposure.

## 4. Discussion

This study demonstrates for the first time by using individual famine exposure data that a relatively short period of moderate or severe undernutrition during childhood is associated with an increase in BMI and waist circumference in adult life, in a dose-dependent manner. Women exposed to famine during their childhood also had an increased risk of being overweight in adult life compared to those who were unexposed.

 Before further discussion, some aspects of our study require consideration. The Dutch famine of 1944-1945 is a “natural experiment” in history, which gave us the unique possibility to study the long-term effects of acute undernutrition during childhood and young adulthood in otherwise well-nourished girls and women. In this study, we used individual data on famine exposure instead of classifying populations according to place of residence or time [28, 29], which we believe to have led to more precise exposure assessment. The drawback of individual data may be their subjective nature, which may have resulted in misclassification. However, misclassification due to recall would most likely have underestimated the observed effects, because we consider it unlikely that recall of famine exposure is related to the risk of overweight. Furthermore, the questionnaires about famine exposure were filled in before height and weight measurements were performed. Our exposure classification data agree with rationing practices at that time. Throughout World War II, the allocated individual amount of calories was based on age. Young children were relatively protected from the famine; children between 1 and 3 years of age received about 50% of the distributed amount of calories at the start of the famine, whereas those aged over 18 years received about 25% [21]. Furthermore, children were relatively protected within families and by special committees such as the Interchurch Organization [21, 30]. These historical facts are reflected in our data; the older the women were at the start of the famine, the higher the proportion that reported to have been exposed to famine. This supports the quality of our exposure data.

 We found a significant dose-dependent increase in BMI and waist circumference in adult life among women exposed to famine during their childhood compared to unexposed women. In agreement with the increase in BMI and waist circumference among these women, we also demonstrated a dose-dependent increased risk of overweight, although the risk of overweight was not significantly increased among moderately famine-exposed women. Adjustment for the potential confounders age at start of the famine, smoking, alcohol intake, and education yielded slightly higher risk estimates for those who reported to be moderately exposed and slightly lower risk estimates for those who reported to be severely exposed. Including total energy intake as a possible intermediate variable into the models, linking childhood undernutrition to later overweight, did not change the results. Thus, the increased BMI, waist circumference, and risk of overweight among famine-exposed women seemed not to be mediated by an increased total energy intake.

 Not only the amount of body fat, which is represented by BMI as a general adiposity measure, but also its distribution is important in evaluating chronic disease risk [31]. Increased abdominal adiposity (visceral fat) has been associated with an adverse metabolic profile and subsequently an increased risk of cardiovascular disease [32]. The results of our study suggest that disturbing important developmental periods during postnatal life are not only associated with general adiposity, but also specifically with abdominal adiposity. These results agree with those of other studies. Rapid infant weight gain after a period of growth restriction has been associated with later abdominal adiposity [33–35]. Among women with anorexia nervosa, weight normalization has been associated with body fat redistribution towards visceral adiposity [36].

Recently, fetal-infant exposure to the Biafran famine has been associated with an increased risk of hypertension, diabetes, and overweight as compared to people born after the famine [37]. However, an association between famine exposure during childhood and the risk of overweight in adult life could not be demonstrated [37]. These results may not seem fully in agreement with ours, but the essentially different circumstances during both famines hamper a direct comparison. The Dutch famine lasted only six months, whereas the Biafran famine lasted for more than two years. Furthermore, the Biafran famine was preceded and followed by periods of relative food shortage instead of adequate nutrition in The Netherlands. The Nigerian standard of living remained rather poor after the Nigerian civil war, whereas the Dutch population grew up in a period of increasing affluence.

Two ecological studies also studied the association between severe undernutrition during childhood and BMI in adult life [28, 29]. Men and women living in Hong Kong who experienced caloric restriction for a continuous period of at least one year during their childhood (around 10 years of age) had a higher BMI in adult life [29]. The other study investigating this association demonstrated that women, but not men, who were exposed to the Great Chinese Famine between 1 and 3 years of age had a significantly increased body weight and BMI and a significantly higher prevalence of overweight and obesity as compared to women who were born after the famine [28]. Since these studies did not assess waist circumference, it is not clear whether the increases in body weight were due to increased abdominal fat mass. In these previous studies, famine exposure was defined by classifying populations according to place of residence. In contrast, our study relied on an individual famine exposure score to define the severity of famine exposure, which we believe to have led to more precise exposure assessment.

### 4.1. Relevance

Our findings support the notion that disturbed growth during postnatal development, particularly in childhood, can have important implications for adult health. The contemporary relevance of our finding is that famine and undernutrition are still a major problem worldwide; never before have there been so many hungry people worldwide [38]. The first Millennium Development Goal is to eradicate extreme hunger. Since childhood overnutrition is also an important precursor for overweight and obesity in adulthood [2], fighting under- and overnutrition of young children may be a powerful strategy to prevent a significant number of deaths due to overweight and obesity at adult age.

### 4.2. Conclusion

This study provides the first direct evidence, using individual famine exposure data, that a short period of moderate or severe undernutrition, especially during early childhood, increases the risk of overweight in adult life.

## Figures and Tables

**Table 1:** Baseline characteristics of the study population according to age at famine (0–9 years, 10–17 years, or ≥18 years) and level of famine exposure (none, moderate, or severe).

	Age at famine								
	0–9 years			10–17 years			≥18 years		
	Level of famine exposure			Level of famine exposure			Level of famine exposure		
	None	Moderate	Severe	None	Moderate	Severe	None	Moderate	Severe
Number (%)	2148 (49)	1612 (36)	665 (15)	1345 (42)	1247 (39)	587 (19)	182 (37)	219 (45)	86 (18)
General characteristics									
Age at start of the famine (years)^a^	4.2 (0–10)	4.6 (0–10)	5.5 (0–10)	13.8 (10–18)	14.1 (10–18)	13.6 (10–18)	18.8 (18–21)	19.1 (18–21)	19.2 (18–21)
Age at recruitment (years)^a^	54.9 (49–63)	55.2 (49–63)	56.2 (49–63)	64.6 (59–70)	64.8 (59–70)	64.0 (59–70)	68.9 (67–70)	69.0 (67–70)	69.1 (67–70)
Body size									
Height (cm)^b^	165.4 (5.9)	165.1 (5.8)	164.9 (6.1)	163.4 (5.7)	163.0 (5.9)	163.1 (6.0)	161.9 (6.2)	162.7 (5.9)	161.3 (6.4)
Dietary intake									
Total energy intake (kcal)^b^	1829 (436)	1817 (428)	1811 (450)	1740 (423)	1750 (405)	1677 (417)	1703 (435)	1663 (400)	1659 (524)
Total protein intake (g)^b^	73.7 (18.4)	72.6 (17.5)	72.9 (18.4)	70.4 (17.0)	70.0 (17.2)	67.5 (16.8)	69.0 (17.1)	68.9 (18.1)	67.6 (22.2)
Total fat intake (g)^b^	72.9 (22.9)	72.3 (22.6)	72.4 (24.1)	68.3 (22.0)	69.6 (21.8)	65.8 (21.9)	67.3 (23.9)	65.1 (20.5)	64.4 (25.1)
Total carbohydrate intake (g)^b^	201.6 (53.5)	201.3 (52.0)	200.5 (53.4)	199.0 (52.7)	198.4 (51.1)	191.8 (52.2)	194.2 (50.3)	190.1 (48.7)	193.5 (65.8)
Lifestyle									
Education in 3 categories^c^									
Low	934 (43)	639 (40)	311 (47)	788 (59)	645 (52)	306 (52)	123 (68)	114 (52)	52 (60)
Intermediate	832 (39)	659 (41)	256 (38)	450 (33)	456 (37)	228 (39)	50 (27)	79 (36)	23 (27)
High	377 (18)	313 (19)	98 (15)	104 (8)	145 (12)	53 (9)	9 (5)	26 (12)	11 (13)
Smoking (pack years)^b^	6.0 (9.2)	6.6 (9.4)	8.1 (10.5)	5.4 (9.4)	6.7 (10.9)	8.0 (11.7)	3.1 (6.1)	6.2 (9.6)	6.2 (10.3)
Alcohol intake (g/day)^b^	10.1 (1.3)	10.1 (1.3)	9.3 (1.3)	6.7 (1.0)	7.1 (1.0)	6.7 (1.2)	6.3 (1.0)	5.9 (9.3)	5.0 (7.0)

^a^Median (min-max), ^b ^Mean (SD), ^c^Number (%).

**Table 2:** *BMI*: means, (un)adjusted differences, and 95% confidence intervals (CIs) for women of all ages combined and within each of the three exposure age categories: 0–9 years, 10–17 years, and ≥18 years who reported to be moderately or severely exposed to famine compared to those who reported to be unexposed to famine.

	Number of subjects	Mean (SD)	Crude model		Multivariable model 1		Multivariable model 2	
			Mean difference^†^	95% CI	Mean difference^†^	95% CI	Mean difference^†^	95% CI
All ages								
Unexposed	3.672	26.0 (4.0)	reference	—	reference	—	reference	—
Moderately exposed	3.074	26.3 (4.0)	0.28	0.09 to 0.48	0.29	0.10 to 0.48	0.34	0.14 to 0.55
Severely exposed	1.335	26.3 (4.2)	0.32	0.07 to 0.57	0.21	−0.05 to 0.46	0.25	−0.02 to 0.52
* P* for trend			0.002		0.02		0.009	
Age at famine categories								
0 to 9 year*s *								
Unexposed	2.147	25.6 (3.9)	reference	—	reference	—	reference	—
Moderately exposed	1.609	25.9 (4.0)	0.25	−0.00 to 0.51	0.33	0.07 to 0.59	0.37	0.10 to 0.64
Severely exposed	664	26.2 (4.3)	0.60	0.25 to 0.95	0.48	0.13 to 0.83	0.44	0.07 to 0.82
* P* for trend			<0.001		0.002		0.004	
10 to 17 years								
Unexposed	1.343	26.5 (4.0)	reference	—	reference	—	reference	—
Moderately exposed	1.246	26.7 (4.0)	0.18	−0.13 to 0.49	0.23	−0.08 to 0.54	0.31	−0.02 to 0.64
Severely exposed	585	26.4 (4.0)	−0.18	−0.57 to 0.20	−0.12	−0.51 to 0.27	−0.02	−0.44 to 0.40
*P* for trend			0.63		0.91		0.64	
≥18 years								
Unexposed	182	26.9 (4.3)	reference	—	reference	—	reference	—
Moderately exposed	219	27.1 (4.5)	0.18	−0.67 to 1.03	0.49	−0.38 to 1.36	0.41	−0.52 to 1.34
Severely exposed	86	27.1 (3.8)	0.25	−0.86 to 1.35	0.44	−0.67 to 1.56	0.75	−0.42 to 1.93
*P* for trend			0.63		0.34		0.19	

^†^Mean difference as compared to those who reported to be unexposed to famine.

*P* for interaction age at famine * famine exposure: 0.02.

Multivariable model 1: adjusted for age at start of the famine (October 1, 1944), smoking (pack years), alcohol intake (g/day), and level of education (3 categories: low, intermediate, and high).

Multivariable model 2: adjusted for age at start of the famine (October 1, 1944), smoking (pack years), alcohol intake (g/day), level of education (3 categories: low, intermediate, and high), and total energy intake (kcal), among the subgroup of women with reliable energy intake according to Goldberg's equation.

**Table 3:** *Waist circumference:* means, (un)adjusted differences, and 95% confidence intervals (CIs) for women of all ages combined and within each of the three exposure age categories: 0–9 years, 10–17 years, and ≥18 years who reported to be moderately or severely exposed to famine compared to those who reported to be unexposed to famine.

	Number of subjects	Mean (SD)	Crude model		Multivariable model 1		Multivariable model 2	
			Mean difference^†^	95% CI	Mean difference^†^	95% CI	Mean difference^†^	95% CI
All ages								
Unexposed	3.670	83.8 (9.9)	reference	—	reference	—	reference	—
Moderately exposed	3.072	84.7 (10.0)	0.91	0.43 to 1.39	0.75	0.28 to 1.23	1.08	0.57 to 1.60
Severely exposed	1.334	84.9 (10.4)	1.19	0.56 to 1.82	0.65	0.02 to 1.27	0.72	0.03 to 1.41
*P* for trend			<0.001		0.007		0.002	
Age at famine categories								
0 to 9 years								
Unexposed	2.145	82.3 (9.6)	reference	—	reference	—	reference	—
Moderately exposed	1.609	83.0 (9.8)	0.74	0.11 to 1.38	0.84	0.21 to 1.48	1.09	0.40 to 1.77
Severely exposed	664	83.8 (10.5)	1.56	0.71 to 2.41	1.02	0.17 to 1.88	0.86	−0.09 to 1.81
*P* for trend			<0.001		0.004		0.008	
10 to 17 years								
Unexposed	1.343	85.7 (9.9)	reference	—	reference	—	reference	—
Moderately exposed	1.244	86.4 (9.9)	0.69	−0.08 to 1.46	0.72	−0.05 to 1.50	1.22	0.37 to 2.06
Severely exposed	584	85.8 (10.2)	0.15	−0.82 to 1.12	0.23	−0.75 to 1.20	0.44	−0.64 to 1.52
*P* for trend			0.45		0.37		0.13	
≥18 years								
Unexposed	182	87.0 (9.6)	reference	—	reference	—	reference	—
Moderately exposed	219	87.2 (9.9)	0.14	−1.79 to 2.07	0.72	−1.26 to 2.71	0.64	−1.64 to 2.91
Severely exposed	86	87.5 (9.9)	0.46	−2.06 to 2.98	0.73	−1.82 to 3.28	1.84	−1.02 to 4.70
*P* for trend			0.73		0.51		0.22	

^†^Mean difference as compared to those who reported to be unexposed to famine.

*P* for interaction age at famine * famine exposure: 0.07.

Multivariable model 1: adjusted for age at start of the famine (October 1, 1944), smoking (pack years), alcohol intake (g/day), and level of education (3 categories: low, intermediate, and high).

Multivariable model 2: adjusted for age at start of the famine (October 1, 1944), smoking (pack years), alcohol intake (g/day), level of education (3 categories: low, intermediate, and high), and total energy intake (kcal), among the subgroup of women with reliable energy intake according to Goldberg's equation.
